# A potential role of autophagy-mediated vascular senescence in the pathophysiology of HFpEF

**DOI:** 10.3389/fendo.2022.1057349

**Published:** 2022-11-17

**Authors:** Fernanda Sanhueza-Olivares, Mayarling F. Troncoso, Francisco Pino-de la Fuente, Javiera Martinez-Bilbao, Jaime A. Riquelme, Ignacio Norambuena-Soto, Monica Villa, Sergio Lavandero, Pablo F. Castro, Mario Chiong

**Affiliations:** ^1^ Advanced Center for Chronic Diseases (ACCDiS), Faculty of Chemical and Pharmaceutical Sciences, University of Chile, Santiago, Chile; ^2^ Division of Cardiology, Department of Internal Medicine, University of Texas Southwestern Medical Center, Dallas, TX, United States; ^3^ Advanced Center for Chronic Diseases, Faculty of Medicine, Pontifical University Catholic of Chile, Santiago, Chile

**Keywords:** autophagy, HFpEF - heart failure with preserved ejection fraction, vascular senescence, vascular aging, obesity, diabetes, hypertension

## Abstract

Heart failure with preserved ejection fraction (HFpEF) is one of the most complex and most prevalent cardiometabolic diseases in aging population. Age, obesity, diabetes, and hypertension are the main comorbidities of HFpEF. Microvascular dysfunction and vascular remodeling play a major role in its development. Among the many mechanisms involved in this process, vascular stiffening has been described as one the most prevalent during HFpEF, leading to ventricular-vascular uncoupling and mismatches in aged HFpEF patients. Aged blood vessels display an increased number of senescent endothelial cells (ECs) and vascular smooth muscle cells (VSMCs). This is consistent with the fact that EC and cardiomyocyte cell senescence has been reported during HFpEF. Autophagy plays a major role in VSMCs physiology, regulating phenotypic switch between contractile and synthetic phenotypes. It has also been described that autophagy can regulate arterial stiffening and EC and VSMC senescence. Many studies now support the notion that targeting autophagy would help with the treatment of many cardiovascular and metabolic diseases. In this review, we discuss the mechanisms involved in autophagy-mediated vascular senescence and whether this could be a driver in the development and progression of HFpEF.

## Cardiovascular diseases and HFpEF

Cardiovascular diseases (CVDs) are the leading cause of death worldwide, resulting in 17.9 million deaths in 2019 (1). On top of the list of most prevalent CVDs is heart failure (HF), a progressive condition in which the heart is unable to pump enough blood to the body and provide the required oxygen levels to fulfill its metabolic demands (2). HF represents the end-stage of multiple cardiac injuries linked to cardiovascular diseases and risk factors; therefore, its prevalence has steadily increased during the last decade affecting approximately 1-3% of the total adult population (3). In the USA, HF affects 5.8 million individuals (2.4% of the population) and is the first cause of hospital admission in adult patients, with a readmission rate during the first six months after discharge of 50% (4). According to the ejection fraction (EF), HF can be classified as HF with reduced EF (<40%, HFrEF), HF with mid-range EF (40-50%, HFmrEF) and HF with preserved EF (>50%, HFpEF). Approximately half of the patients with signs and symptoms of HF have HFpEF. Predisposing risk factors for HFpEF include older age, diabetes, obesity, and arterial hypertension (5). Even though these syndromes show similar symptoms (edema, dyspnea, fatigue, exercise intolerance), it is well described in the literature that they are quite different syndromes: HFpEF is characterized by diastolic dysfunction, altered ventricular relaxation and filling, increased stiffness, and concentric remodeling of the ventricular wall, resulting in an important pressure overload. On the other hand, HFrEF is characterized by systolic dysfunction, altered ventricular contraction, which reduces ejection fraction, and an eccentric myocardial remodeling followed by ventricular dilation, resulting in ventricular volume overload (2). Compared to HFrEF, HFpEF presents with increased cardiac perivascular fibrosis, less nitric oxide (NO) bioavailability, earlier endothelial dysfunction and higher level of pro-inflammatory cytokines (6). Additionally, HFpEF patients present a higher load of comorbidities, mainly advanced age, obesity, diabetes and hypertension (7, 8). Dunlay et al., (3) reported a summary of an important number of clinical trials using interventions well described for HFrEF that have shown little or no effect on mortality rates in patients with HFpEF, underlying the importance of the study and development of new therapeutic strategies to treat HFpEF.

HFpEF is associated with a poor quality of life, crucial healthcare resource utilization, high rates of hospitalization, and mortality that are similar to patients with HFrEF (9). Incidences of obesity and diabetes mellitus are projected to grow, leading to an increased prevalence of risk factors for HFpEF (10, 11). HF prevalence of both types increases with age, but the prevalence of HFpEF at any given age increases more rapidly than HFrEF prevalence (12). One of the main limitations of early studies in HFpEF was the lack of established diagnostic criteria (13). In many registries, patients diagnosed with HFpEF also had other comorbidities that could account for their symptoms, such as extreme obesity, lung disease, and myocardial ischemia (13). It was only recently that The European Society of Cardiology proposed a comprehensive set of diagnostic criteria. These criteria allowed for identifying patients with HFpEF, ruling out confounding comorbidities, and reaffirming the existence of the clinical problem (14).

HFpEF is considered a clinical syndrome rather than a discrete disease (9). Therefore, multiple pathophysiological mechanisms, including diastolic dysfunction, are responsible for its generation (9, 15). In recent years, chronic systemic microvascular inflammation has been highlighted as one of the main pathophysiological mechanisms of HFpEF, and the importance of the different comorbidities that induce this response, mainly age, obesity, diabetes, and hypertension, has been emphasized (7, 16, 17). Patients with HFpEF show elevated inflammatory markers such as Interleukin-1 type I receptor (IL-1R), tumor necrosis factor α (TNFα), C-reactive protein (CRP), vascular cell adhesion molecule-1 (VCAM-1) and IL-6 (7, 15). This leads to increased endothelial reactive oxygen species (ROS) production, less NO bioavailability, and nitrosative stress due to the accumulation of nitrogen reactive species (RNS) (7, 18).

In this review, we go through available data regarding vascular and microvascular dysfunction related to autophagy and cell senescence, to propose a potential role of these processes in the development and progression of HFpEF.

## Vascular contribution to HFpEF

### Microvascular dysfunction

The microvasculature is comprised of arterioles, capillaries and venules; microcirculation through these vessels allows the delivery of oxygen and nutrients to meet the energetic demands of local tissues, mainly through regulation of vascular tone, structural microvascular adaptations such as angiogenesis, and the regulation of hemostasis, inflammation and vascular permeability (19, 20). The role of microvascular dysfunction, usually described as an impaired regulation of blood flow in response to oxygen requirements (21), has been widely described in several chronic conditions, such as hypertension, diabetes, obesity, and HF (22). As previously mentioned, Paulus and Tschope (7) proposed a new paradigm for HFpEF in which myocardial dysfunction is, in part, due to coronary microvascular inflammation. They proposed that low-grade chronic systemic inflammation, mainly because of the presence of multiple comorbidities, initiates detrimental microvascular changes that result in the HFpEF-associated myocardial dysfunction. Accordingly, inflammatory markers such as IL-6, CRP and TNFα showed a stronger association in HFpEF patients when compared with HFrEF patients (23). In summary, it is proposed that inflammation induces ROS production in the endothelium, which decreases NO bioavailability and increases peroxynitrite levels, impairing the guanylate cyclase/cyclic guanylate monophosphate (cGMP)/protein kinase G (PKG) pathway in cardiomyocytes (24). This was further described in cardiomyocytes of a mice model of HFpEF treated with high fat diet (HFD) and N(ω)-nitro-L-arginine methyl ester (L-NAME), a nitric oxide synthase (NOS) inhibitor (18). Whether the same effects occur in VSMCs is yet to be determined. Additionally, HFpEF patients show systemic peripheral impaired microvascular reactivity evaluated by endoPAT, a non-invasive endothelial dysfunction test (25), and capillary rarefaction in skeletal muscle associated with poor exercise tolerance (26). Moreover, a decrease in coronary microvascular function, an increased prevalence in coronary rarefaction, and an impaired maximal hyperemia, compared to age-matched controls, were also reported in HFpEF patients (25–28). For a more comprehensive review on this subject, see Weerts et al., (29).

Recently, using cell therapy, de Couto et al., (30) administered intracoronary cardiosphere-derived cells (CDCs) during two weeks to HFpEF rats, developed by feeding Dahl salt-sensitive rats with a high salt diet. CDCs treatment improved EC-dependent vasodilation, reduced oxidative stress, restored endothelial NOS (eNOS) expression, previously shown to be decreased in the HFpEF endothelium (31), inflammatory response and VCAM-1 expression. It also improved diastolic dysfunction and restored vascular reactivity (30). These results uncover the importance of microvascular dysfunction in HFpEF. Another approach to evaluate microvascular function is the study of the retinal arterioles, which can be evaluated using non-invasive optic cameras (32). Retinal arterioles wall-to-lumen ratio (rWLR) was significantly higher in HFpEF group, consistent with a previously proposed correlation between retinal arteriole structural alterations and HF (33). Interestingly, this increase was also significant when compared to hypertensive controls (32). Finally, a recent study performed by Yuksel et al., (34), evaluated microvasculature using nailfold videocapillaroscopy in HFpEF patients and found abnormal results regarding microvascular morphology, architecture and density compared not also with control patients but also with patients with HFrEF.

**Figure 1:**
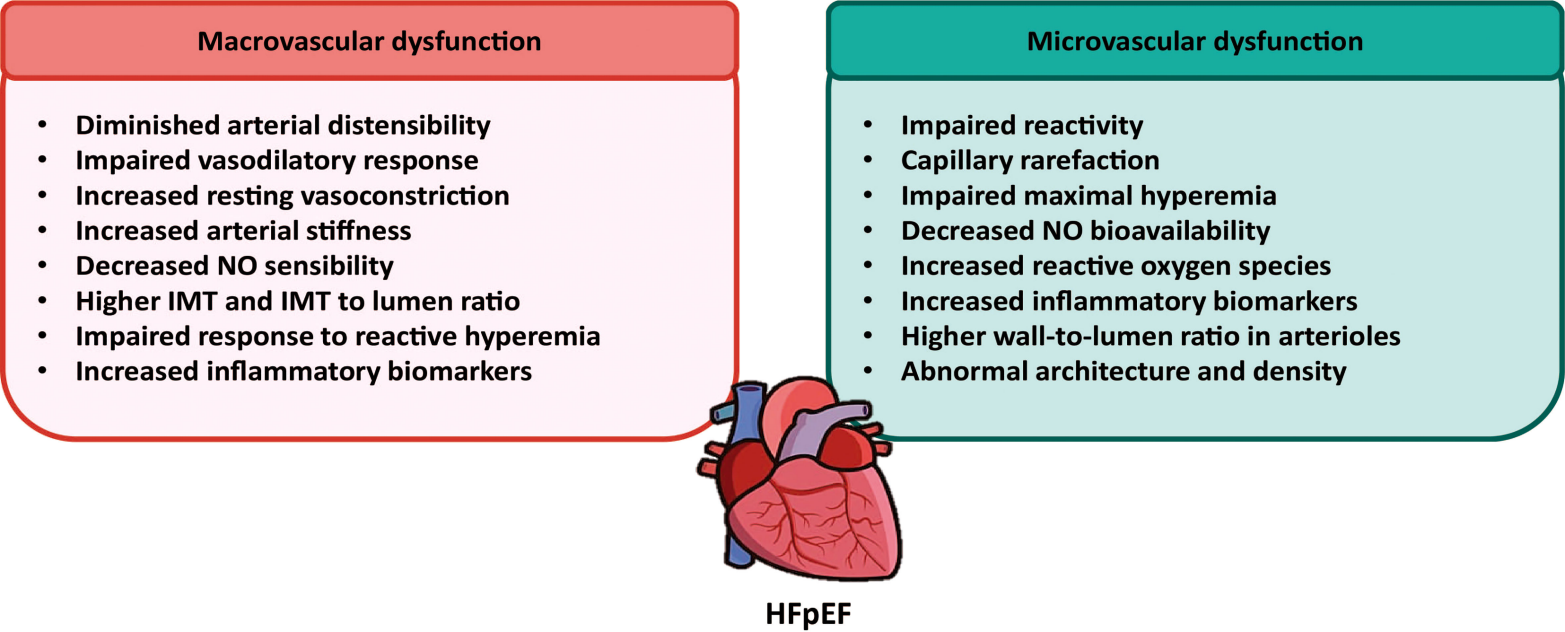
Contribution of macrovascular and microvascular dysfunctions to the development of HFpEF. Using non-invasive techniques, several macro and microvascular alterations have been described in the resistance and conduit arteries from HFpEF patients. These alterations are suggestive for a main role of vascular dysfunction in the pathogenesis of HFpEF.

Taken together, the evidence presented above strengthens the previously proposed role of microvascular dysfunction in HFpEF pathogenesis (**Figure 1**). However, remains to be elucidated whether this microvascular dysfunction is cause or consequence of HFpEF.

### Macrovascular dysfunction

Arterial intima-media thickness and stiffness have been identified as important risk factors for HF (35). The assessment of major vessel function in HFpEF started with aorta analyses from HFpEF patients. These aortas present a diminished aortic distensibility (36), a decreased vasorelaxation response induced by nitroglycerine (37) and exercise (38, 39), increased resting vasoconstriction and less NO sensibility (17). Furthermore, an increased aortic stiffness has been widely reported in these patients (17, 40–42). Schwartzenberg et al., (43) reported that HFpEF patients responded better to treatment with sodium nitroprusside (a vasodilator drug), as compared with HFrEF patients, underlying the importance of the vascular component to HFpEF. Among the mechanisms that relate arterial stiffening to myocardial dysfunctions are an impaired myocardial oxygen supply, reduced arterial compliance and a decrease in diastolic blood pressure, that compromise coronary artery flow (44).

An association between HFpEF carotid arteries morphological and functional alterations has been recently described (32, 45, 46). Using ultrasound and magnetic resonance imaging (MRI) on patients from the multi-Ethnic study of atherosclerosis (MESA), Fernandes et al., (44) found a relation between carotid distensibility and enhanced left ventricle diastolic function, even after adjustment for risk factors and blood pressure control therapies. In another study that analyzed MESA patients, no difference in the association of carotid intima-media thickness (IMT) was reported when comparing HFpEF with HFrEF patients (46). Additionally, after adjustment with traditional risk factors, the observed association of internal and common carotid IMT and HF lost its statistical significance. Moreover, it was recently described that common carotid arteries of HFpEF patients had a higher diameter IMT and a significantly higher IMT to lumen ratio compared to controls (32). Interestingly, blood pressure control decreases carotid IMT but did not stop its progression in hypertensive subjects (47). Engstrom et al., (48), reported that there was a significant association of carotid IMT and HF hospitalization. Through the evaluation of brachial-ankle pulse wave velocity (baPWV), a method to estimate arterial stiffness, which reflects the stiffness of both the aorta and peripheral artery, Hu et al., (49), described a positive correlation between baPWV and left ventricular hypertrophy and diastolic function. Similarly, using brachial artery flow-mediated dilation (FMD) and microvascular function *via* reactive hyperemia (RH), it was reported that HFpEF patients had a reduction in brachial artery diameter in response to RH, compared to aged-matched controls (16). Nevertheless, this difference was lost when normalizing for shear stress rate differences. In accordance with the latter, no differences were found between HFpEF patients’ femoral artery FMD and those of the controls (50). On the other hand, a totally different result was described by Farrero et al., (51), in which a significant reduction in brachial artery FMD was observed in HFpEF patients compared with hypertensive controls. To further support the importance of the vasculature to HFpEF pathophysiology, several biomarkers related with vascular function are altered during HFpEF: reduced NO bioavailability (potent vasodilator), higher levels of endotelin-1 (a potent vasoconstrictor) and increased levels of plasminogen activator inhibitor-1 (PAI-1), a described risk factor for atherosclerosis, among others (52).

It is important to note that most of the studies discussed in this section have been performed using non-invasive methods, such as MRI, FMD, and ultrasound. Additionally, human studies are usually performed with patients taking medication for HFpEF comorbidities, mainly anti-hypertensive drugs, which limits the data obtained about the natural history of the disease. Very little research focusing on molecular alterations have been performed, especially in relation to macrovascular circulation. This may be due to the, until recently, lack of animal models that properly emulate HFpEF characteristics. In the past few years, an important number of animal models have been developed, so further investigation will hopefully reveal more detailed information about vascular dysfunction and remodeling during HFpEF onset and progression. Even though there is a long way to go to fully elucidate the molecular mechanisms behind vascular alterations in HFpEF, it is now clear that micro and macrovascular dysfunctions could play a major role in HFpEF (**Figure 1**).

## Vascular aging and senescence

The mechanisms of aging and age-associated disorders are complex and involve cellular senescence (53). Aging is characterized by an accumulation of senescent cells, as a result of aged organisms being unable to repair damage at the same rate as cells become damaged (54). Senescent cells can be identified by a permanent cell growth arrest (55, 56). Senescent cells become enlarged and flattened, with proliferative arrest, that secretes pro-inflammatory molecules, a phenotype known as senescence-associated secretory phenotype (SASP), triggering chronic sterile inflammation that induces tissue remodeling (53). Several markers are used to indirectly detect senescent cells, being senescence-associated β-galactosidase (SA β-gal) activity the most common. Lysosomal β-gal activity is detected at a low pH (around pH 4) but becomes detectable at a higher pH (pH 6) in senescent cells due to marked expansion of the lysosomal compartment (57). Other markers of cellular senescence include high expression of p53, p16, p21, p38-mitogen activated protein kinase (MAPK), and phosphorylated histone H2AX (γH2AX), an early marker of cellular response to the induction of DNA double-strand breaks (58–62). Moreover, high mobility group A (HMGA) proteins and heterochromatin markers, including heterochromatin protein-1 and tri-methylated lysine 9 histone H3, are molecular markers of senescence-associated heterochromatin foci and are considered to reveal cellular senescence (61).

Arterial remodeling occurs with aging, even in the absence of cardiovascular disease and cardiovascular risk factors. Aging is frequently associated with vascular dysfunction (63, 64). In fact, people with progeria syndrome, that present a premature aging in early childhood, developed premature atherosclerosis disease (65). Aged arteries have increased intima/media thickness ratio, with an increase of 2- to 3-fold from 20 to 90 years of age (66, 67). The arterial media also becomes thicker with aging, and its cellularity decreases simultaneously (68). Furthermore, the length and circumference of the aorta increase with aging (69), with an accumulation of collagen and elastin decline (70). These structural changes are associated with a reduction in compliance, reduction of elasticity/distensibility, and increase of stiffness, resulting in higher systolic blood pressure and lower diastolic pressure (71). ROS and chronic low-grade sterile inflammation are two significant contributors to the progression of age-related vascular dysfunction. Senescent cells accumulate in the arteries with aging irrespective of whether a person has or not age-related vascular disorders (72–75). Along with aging, vascular tissues of rodents and humans show elevation of the levels of p16, p21, phosphorylated p38-MAPK, and double-stranded DNA breaks, in association with high SA β-gal activity (76–79). Expression of p53 and p21 is increased in the arteries of elderly persons, together with a structural breakdown of telomeres (75). Interestingly, senescent cells are increased in the coronary arteries of patients with ischemic heart disease but not in the internal mammary arteries (72).

Blood vessel walls are comprised primarily of ECs, VSMCs and extracellular matrix (ECM). Because in both cells are described the occurrence of phenotypic features commonly observed in senescent cells (74), and changes in vascular ECM structure is associated with aging (80), the association of these 3 vascular components with senescence and CVDs is next described.

### Vascular smooth muscle cells

VSMCs are the key component of the medial layer in arteries, with an important role in contraction and regulation of blood pressure and vascular tone (81). Aging-dependent functional changes of VSMCs are partly due to deregulation of TGF-β signaling, and these cells undergo a transformation from “contractile” to “synthetic” phenotype (82, 83). The VSMC synthetic phenotype is responsible for the aging-dependent intimal thickening because of the increased proliferation, migration and production of collagen (70). Moreover, upon metabolic alterations, the shift from a contractile to a synthetic phenotype has been associated with progression of hypertension and atherosclerosis (84).

Intimal thickening is also associated with the formation of atherosclerotic lesions (85). Interestingly, senescent VSMC have been identified in atherosclerotic lesions of patients with coronary artery disease and peripheral artery disease (72). Those VSMC present shorter telomeres, are positive for SA β-gal, and have elevated p16 and p21 expression (74, 86). Senescent VSMC in atherosclerotic plaque display loss of telomeric repeat-binding factor-2 (TRF2). TRF2 overexpression reduces DNA damage, accelerates DNA repair, and suppresses cellular senescence (87). VSMC specific knockout (KO) of TRF2 increases atherosclerosis and necrotic core formation. These pathological changes are inhibited in mice with VSMC-specific overexpression of TRF2 (87). Hypertension, an established risk factor for HFpEF, increases the activity of p53 and p21 in the arteries of hypertensive patients. While telomere length is comparable between patients with hypertension and controls, telomere uncapping is 2-fold higher in hypertensive patients (88). A murine model of genomic instability showed senescence of ECs and VSMC in the aorta, along with impaired vasodilation, increased vascular stiffness, and hypertension (89). In a hypertensive rat model, produced by treating with deoxycorticosterone acetate and salt, overexpression of p16 was detected in the coronary arteries, indicating the existence of a vicious circle between cellular senescence and hypertension (90). Another vicious circle is produced because senescent VSMCs trigger low grade sterile inflammation through the secretion in the SASP of several cytokines, including IL-1α (91). Moreover, SA β-gal positive VSMC in carotid plaques express IL-6, suggesting that senescent VSMCs have a SASP involved in the progression of atherosclerotic disorders (91). Taken together, these studies show that senescent cells accumulate in the vessels of patients with atherosclerosis, hypertension, aneurysms, and intimal hyperplasia, some of them common risk factors for the development of HFpEF.

ROS and angiotensin (Ang) II are well-known inducers of senescence in VSMC (92, 93). Ang II administration also induces senescence of VSMCs in apolipoprotein E null mice (94). Ang II promotes VSMC senescence by suppressing Mdm-2-mediated degradation of p53 and promoting the expression of smooth muscle 22α (SM22α) (95). Similarly, we described that Ang II increases contractile proteins calponin and α-smooth muscle actin (α-SMA) (96). ROS induces DNA damage in VSMC and suppresses telomerase activity, leading to telomere shortening and cellular senescence in the atherosclerotic lesion (86). On the other hand, hypoxia inhibits senescence by promoting telomerase activity (97).

As reviewed by Chi et al., (98), epigenetics can accelerate or prevent VSMC senescence. Autophagy, the mechanism by which cells removes damaged components (99) could also prevent VSMC senescence as vascular aging is associated with impaired autophagy (100) and induced moderate autophagy can increase proliferation in VSMCs (101). Although it is possible to detect senescence in VSMCs using common senescence criteria, such as changes in levels of p16, p21, p38-MAPK, p53 and H2A.X and SA β-gal activity (102), in a study aimed to characterize human coronary VSMC senescence it was concluded that classical senescence markers show a mild deregulation as to warrant consistent senescence detection *in vitro*, and that altered RNA metabolism could be a key feature to ensure VSMC senescence detection (103).

### Endothelial cells

The endothelium is a semipermeable barrier composed by a monolayer of cells that controls the exchange of nutrients and metabolites, regulates vascular tone, permeability, inflammation and blood fluidity and, thereby, is of paramount importance for maintaining vascular homeostasis (104, 105). ECs are a constant target for different damage-inducing factors present in the bloodstream that may impair cellular function. EC senescence can occur as a result of this cell damage, thereby precluding uninhibited proliferation of damaged cells, but this process can also be harmful and contribute to the pathophysiology of cardiovascular diseases (106). During aging, ECs display the classic markers of cell senescence (64). Additionally, alterations in mitochondrial biogenesis (107), NFkB activation (108), increased matrix metalloproteinase (MMP) secretion (109) and reduced eNOS activity (110) have also been described. In human umbilical vein ECs (HUVECs), knockdown of the transcription factor E2F2 induced senescence in these cells and its overexpression decreased senescence markers; interestingly, a lower expression of E2F2 was seen in aortas of aged mice (111). These experiments suggest that E2F2 can be a potential target to modulate senescence *in vivo*, yet its participation in HFpEF remains unknown. Finally, during aging, Sirtuins dysregulation occurs in the endothelium, particularly a decrease in SIRT1, which induces cell senescence and has been linked to the development of CVDs, as reviewed by Kida and Goligorsky (112). Nevertheless, Conti et al., (113) found no significance differences in SIRT1 levels in peripheral blood mononuclear cells of HFpEF patients compared to controls. Whether these differences are due to the different cell type studied or not requires to be studied.

### Vascular extracellular matrix

The ECM is composed of several structural proteins, including elastin and collagens, that not only provides structural support to the VSMCs and ECs, but also regulates the mechanical function of the vessel (80). Arteries stiffen with age, suggesting that age-related arterial stiffening may contribute to CVDs (114). In fact, elastin fibers lose functionality with age mainly by fragmentation, calcification and MMP degradation. These changes induce the formation of stiffer fibrils, which directly contributes to age-dependent increases in arterial stiffness (114). In contrast, the arterial collagen content and collagen crosslinking increases with age (80). Increased fibrosis has been described in the intima (115), media (116, 117), and adventitia (118).

Age-related changes in elastin and collagen composition and function are due to the action of MMPs (80). MMPs and its tissue inhibitors (TIMPs) changed as a function of age in the absence of clinically significant CVD (119). In the blood vessels, age-related MMP-2 upregulation occurs in the human aorta but not in the internal mammary artery (120), and this upregulation is associated to arterial stiffness (121). Moreover, MMP-3 polymorphisms have been associated with vascular remodeling and age-related arterial stiffening (122). Several factors that are dysregulated during vascular senescence, such as NO, IL-1 and TNFα, trigger MMPs synthesis and activation (80). Moreover, MMP activation, which disrupts arterial integrity, can be induced by oxidative stress (123). An interesting study showed a ROS-dependent activation of MMPs in cerebral arteries of aged, but not young, hypertensive mice (109). This finding supports the notion that multiple comorbidities, i.e. age and hypertension, are required for some of the pathological alterations observed in these arteries.

ECM alterations create a pro-inflammatory environment that induce phenotypic alterations in both ECs and VSMCs, such as those observed during vascular aging and senescence (124). Since low-grade chronic inflammation has been proposed as one of the key features of HFpEF, it could be assumed that these ECM alterations play an important role in this disease. Although no studies have been performed that evaluate vascular MMP levels and activity in HFpEF, a cardiome-directed network analysis performed in a rat model of HFpEF showed that ECM alterations occur in the heart of these animals (125). Additionally, a correlation between MMP-2 levels and left ventricle EF was found in HF patients (126). In fact, MMP-2 has been proposed as a target for HF treatment (127). Furthermore, human primary fibroblast from patients with both hypertension and HFpEF showed a significant decrease in membrane type 1-MMP, compared with hypertensive only and healthy patients (128). Whether similar alterations occur in vascular tissue remains to be determined.

## Autophagy, cell senescence and vascular aging

Autophagy is a physiological process that seeks to maintain cellular homeostasis by controlling the degradation of components such as proteins and damaged organelles (129). ECs and VSMCs are no strangers to this process, and multiple diseases have been associated with an imbalance in the autophagic flux (130). Among the most described autophagy hallmarks to evaluate this process are the accumulation of p62, LC3-II levels, LC3-II/LC3-I ratio, the analysis of autophagy related protein (Atg) levels and some of the main regulatory proteins such as Beclin-1, ULK1 and mTOR (131). The relationship between senescence and autophagy has been explored in both VSMCs and ECs.

### Vascular smooth muscle cells

An increase in autophagy has been related to phenotypic changes in VSCMs from a differentiated to a dedifferentiated one, which favors the appearance of different CVDs (130). On the other hand, autophagic flux blockage has also been related to a phenotype change in diseases such as aneurysms and atherosclerosis (132). A direct link between VSMC autophagy and arterial stiffness is demonstrated using a VSMC-specific Atg7-KO mice (Atg7F/F SM22α-Cre+ mice) (133). Moreover, specific deletion of Atg7 in VSMC induces p62 accumulation and accelerates the development of stress-induced premature senescence (134). During aging, an impaired autophagy is triggered due to direct oxidation of Atg3 and Atg7 that inhibits LC3 lipidation (135) and due to mTOR activation (136). Also, an increase of IL-6 and impairment of mitochondrial function within the aorta, associated with enhanced mitophagy and increased PARKIN levels are observed (137). Furthermore, during aging, the expression of Krüppel-like family of transcription factor 4 (KLF4) decreases in vascular tissues in C. elegans, mice and humans (138). Overexpression of KLF4 increases autophagy flux and improves vessel function in aged mice, suggesting an evolutionary transcriptional regulation of autophagy during aging (138). Accordingly, activation of autophagy by the upregulation of the peroxisome proliferator activated receptor gamma coactivator 1 alpha (PPARGC1A) (139), celastrol (a quinone methide triterpenoid isolated from the Celastraceae family) (140), genistein (141) or nifedipine (142) suppresses VSMC senescence by upregulating autophagic flux.

In both replicative and stimulus-induced *in vitro* senescence models, it has been demonstrated that autophagy is required for VSMC senescence development. In rat VSMC treated with Ang II, an increase in SA β-gal activity, p16, p21, and p53 levels are observed (143, 144). Treatment with Ang II also decreased VSMC proliferation (140). Interestingly, these studies show a decrease in autophagic flux, so using an autophagy inducer such as rapamycin, Ang II-induced senescence is prevented (140, 143). Doxorubicin was also shown to induce VSMC senescence through an autophagy-dependent mechanism. A decreased autophagic flux through activation of mTOR and downregulation of essential autophagy proteins such as Beclin-1 and LC3 were described in these cells (136, 141). Other VSMC senescence inducers have been shown to have a slightly different mechanism. For example, hydrogen peroxide has been shown to induce senescence by blocking autophagic flux, causing LC3 accumulation (142). On the other hand, oxLDL induces senescence but does not produce modification of LC3 levels or ULK1/mTOR phosphorylation. Despite this, rapamycin prevents oxLDL-induced VSMC senescence (101). This proves that even when autophagy is not involved directly in the induction of cell senescence, it can be a rescue mechanism.

In both human and rat VSMC replicative senescence models, increased mTOR signaling is observed, but upon treatment of these cells with rapamycin, all senescence markers are decreased (145). In aged cells, a decrease in Beclin-1 and LC3 levels and an increase in mTOR phosphorylation are also observed. Remarkably, upon increasing autophagic flux, senescence is reversed (146). Finally, Grootaert et al., (134) showed that Atg7-KO VSMC displayed lower proliferation rate and increased senescence markers, consistent with an acceleration of senescence. Although there are some differences in the molecular mechanisms of the different treatments and the time of treatment to induce senescence, the same pattern is observed in all of them; autophagy inhibition is an essential step in the induction of senescence in VSMCs. Therefore, current evidence supports the idea that autophagy activation in VSMC could have protective effects in the aging-associated development of CVDs.

### Endothelial cells

In general terms, autophagy is crucial to maintain the homeostasis of ECs, while impaired autophagic flux can lead to endothelial inflammation and thereby, favor the development of atherosclerosis (147). Recent studies have delved into the role of endothelial autophagy in CVDs. Gogiraju et al., (148) reported that mice lacking the endothelial leptin receptor and subjected to transverse aortic constriction showed improved left ventricular function and reduced hypertrophy. Moreover, deletion of the leptin receptor was associated with increased autophagy and impairment of the Akt/mTOR pathway, suggesting a protective role for autophagy in a pressure overload setting, which is suppressed by leptin signaling (148). Another study reported that mice with EC-specific deletion of autophagy-related protein 7 (Atg7) show increased susceptibility of doxorubicin-induced cardiotoxicity (149). These data further support a protective role for autophagy in CVDs.

The link between endothelial autophagy and senescence has also been explored. Rhynchophylline has been found to reduce Ang II-induced senescence *via* AMPK-dependent activation of autophagy in endothelial progenitor cells (150). In addition, C1q/tumor necrosis factor-related protein 9 (CTRP9), which wields anti-aging and anti-atherogenic effects, was recently found to reduce endothelial senescence induced by palmitic acid in HUVECs, an effect also achieved through AMPK-mediated activation of autophagy (151). Pan et al., (152) showed that the overexpression of Yes-associated protein (YAP) in HUVECs and in rat aortas increased the activity of SA β-gal staining and protein markers such as p16, p21 and p53, along with an activation of mTOR pathway and a blockage of autophagic flux. They also demonstrated that the knockdown of YAP and the inhibition of mTOR could relieve both cellular and vascular senescence (152). Advanced oxidation protein products (AOPPs) result from cell oxidative stress and are accumulated and increased in patients with vascular disease and aging (153). In HUVECs, AOPPs induced senescence, increasing the expression of p21, p16 and SA β-gal activity, along with an impairment in autophagic flux (154). The effects of AOPPs were also evaluated in a model of ApoE^-/-^ mice fed with a HFD, which showed an increase in senescence molecular markers in aortic tissue (154). Interestingly, it has been reported that autophagy reduces apoptosis and senescence induced by high glucose concentrations in human coronary artery ECs (155). Nonetheless, a protective role for endothelial autophagy and its potential effect in cellular senescence in the context of HFpEF remains to be elucidated.

## Autophagy and HFpEF

The role of autophagy is well described in HFrEF (156). Although its role in HFpEF is poorly described, there is evidence that autophagy could play an important role in the development of HFpEF. cDNA analysis in a rat model showed that five processes are mainly involved in the development of HFpEF: endothelial function, inflammation, sarcomere/cytoskeleton, extracellular matrix and apoptosis/autophagy (125). RNAseq analysis performed in patients with HFpEF also revealed that genes related to endoplasmic reticulum stress, angiogenesis, and autophagy, are related to the development of HFpEF (157). Similarly, in a model of aged HFpEF mice, through a RNAseq analysis, it was found that the most upregulated pathways were those related to cell cycle and mitotic cell cycle processes in the heart of aged mice (158). Animal studies show that both autophagy and mitophagy decrease in the heart with age (159, 160). LC3-II expression and the LC3-II/LC3-I ratio decrease in the heart of aged mice with diastolic dysfunction, compared to young controls (159). On the other hand, myeloid differentiation protein 1 (MD1) is decreased in the heart of HFpEF mice and its down regulation promotes autophagy through a ROS/MAPK pathway (161). Mitochondrial dysfunction is one of the central mechanisms in the development and progression of HF (162). In this line, mitophagy was found to be decreased in aged mice. Interestingly, p53 was found to inhibit PARKIN translocation into the mitochondria, decreasing mitophagy and thus contributing to mitochondrial dysfunction. In addition, p53-KO mice improved mitochondrial integrity and cardiac functional reserve in both aged and doxorubicin-treated mice. PARKIN overexpression also improved cardiac function and decreased SA β-gal activity (160). These data highlight the importance of autophagy to be recognized as a target to be studied in development and treatment of HFpEF.

## Senescence and HFpEF

EC senescence has been described to play an important role in aging mice with HF (163). The presence of comorbidities associated with age such as hypertension, diabetes or obesity contribute to endothelial inflammation and the consequent reduction of its ability to induce vasodilation, which in turn elicits cardiac hypertrophy, stiffness and ultimately, HF (7, 106). Chronic sterile inflammation, probably due to a SASP, is present in the myocardium of HF patients (164), and is involved in the induction of cardiac remodeling (165). In a murine model of left ventricular pressure overload, cardiac and endothelial p53 levels are increased, leading to cardiac inflammation associated with suppression of myocardial angiogenesis, tissue hypoxia, and cardiac dysfunction (166, 167). These studies suggest the involvement of cardiac senescence in the pathophysiology of HF.

One of the major risk factors for HFpEF is age (5, 9). Moreover, it was recently suggested that EC senescence also contributes to the development of HFpEF: when mice with accelerated senescence were fed a high-fat, high-salt diet, both EC senescence and inflammation increased, along with the typical hemodynamic and structural changes of HFpEF and an impaired endothelial-dependent vasodilation of the aorta (163). Furthermore, the histological analysis of thoracic aorta revealed that the pro-inflammatory protein ICAM-1 and the senescence marker acetyl-p53 were increased in ECs of senescence accelerated mice (SAM) fed with Western diet, as compared with the SAM fed with control diet, suggesting that the potential therapeutic targeting of EC senescence may be a valuable strategy for the treatment of HFpEF (163). Using an aged mice model of diastolic dysfunction, Shinmura et al., (159), showed increased levels of SA β-gal of aged mice. An interesting model of telomerase RNA KO plus diet-induced HFpEF (HFD and L-NAME supplemented-water), showed increased p53 expression in the heart, associated with impaired mitochondrial respiration, and that myocardial-specific p53 KO mice show a delay in the development of HFpEF, although the pathology still developed (168). Patients from the multicenter PROMIS-HFpEF study with a pan-inflammatory phenotype had increased levels of insulin-like growth factor-binding protein 7 (IGFBP7), a protein that stimulates inflammation and cell senescence (169). Accordingly, HFpEF patients from the RELAX trial showed a higher baseline IGFBP7 that was correlated with impaired diastolic function (170). Nevertheless, while the use of senolytics is a promising therapeutic approach (171), the evidence linking senescence to HFpEF is still scarce and more studies are required to confirm these findings.

Considering that cellular senescence induces vascular dysfunction and inflammation, it seems reasonable that it would also promote pathologic changes observed in HFpEF. Moreover, as stated above, chronic microvascular inflammation, a senescence-like phenotype, is one of the hallmarks of HFpEF development (7).

## A possible role of vascular autophagy-mediated senescence in HFpEF

So far, we have presented evidence that: 1) microvascular and macrovascular dysfunction are present in HFpEF and play an important role in its pathophysiology, 2) aging, one of the most prevalent comorbidities of HFpEF involves both vascular senescence and vascular autophagy impairment, 3) autophagy modulates cells senescence in both VSMC and ECs and 4) both autophagy and cell senescence in the heart muscle and endothelium are most likely to be participating in the development and progression of HFpEF.

It is currently believed that chronic low-grade inflammation is one of the main drivers of HFpEF, resulting in a decreased NO bioavailability, an increase in proinflammatory cytokine levels and oxidative stress (7). ROS and inflammation have also been described as characteristic features of vascular aging, and the resulting vascular remodeling and dysfunction (172). It has been shown that ROS induces senescence in both VSMCs and ECs (92, 93, 154), and that this leads to unpaired vasodilation and vascular stiffness, both of which have been observed in HFpEF patients (17, 37–42). As with aging, during hypertension, another important comorbidity of HFpEF, vascular senescence is observed (88). Taken together, this data supports the idea that vascular senescence could be an important component of HFpEF physiopathology. IGFBP7, a protein involved in the regulation of cell senescence, was found to be increased in HFpEF patients (169, 170). So far, only one study has been performed that evaluates senescence markers in a diastolic dysfunction mouse model and shows that there is an increase in p53 levels and SA β-gal activity in aortic ECs of these mice (163).

The inhibition of autophagy leads to cell senescence in both VSMCs and ECs (134, 149). On the same line, autophagy induction can prevent the appearance of cell senescence (101). It has also been described that vascular aging is accompanied by an impaired autophagy, which results in vascular stiffness (135). Oxidative stress, mentioned as an important component of HFpEF, induces VSMC senescence through blockage of autophagy (142). Since autophagy is starting to appear as an emerging pathway involved in HFpEF, mainly through genetic analyses, its relationship with vascular senescence is a promising field of study. Hearts of aged mice with diastolic dysfunction show impaired autophagy (159). Nevertheless, no studies have been performed that evaluate autophagy in vascular tissue during HFpEF, nor its relationship with cell senescence.

## Concluding remarks

**Figure 2:**
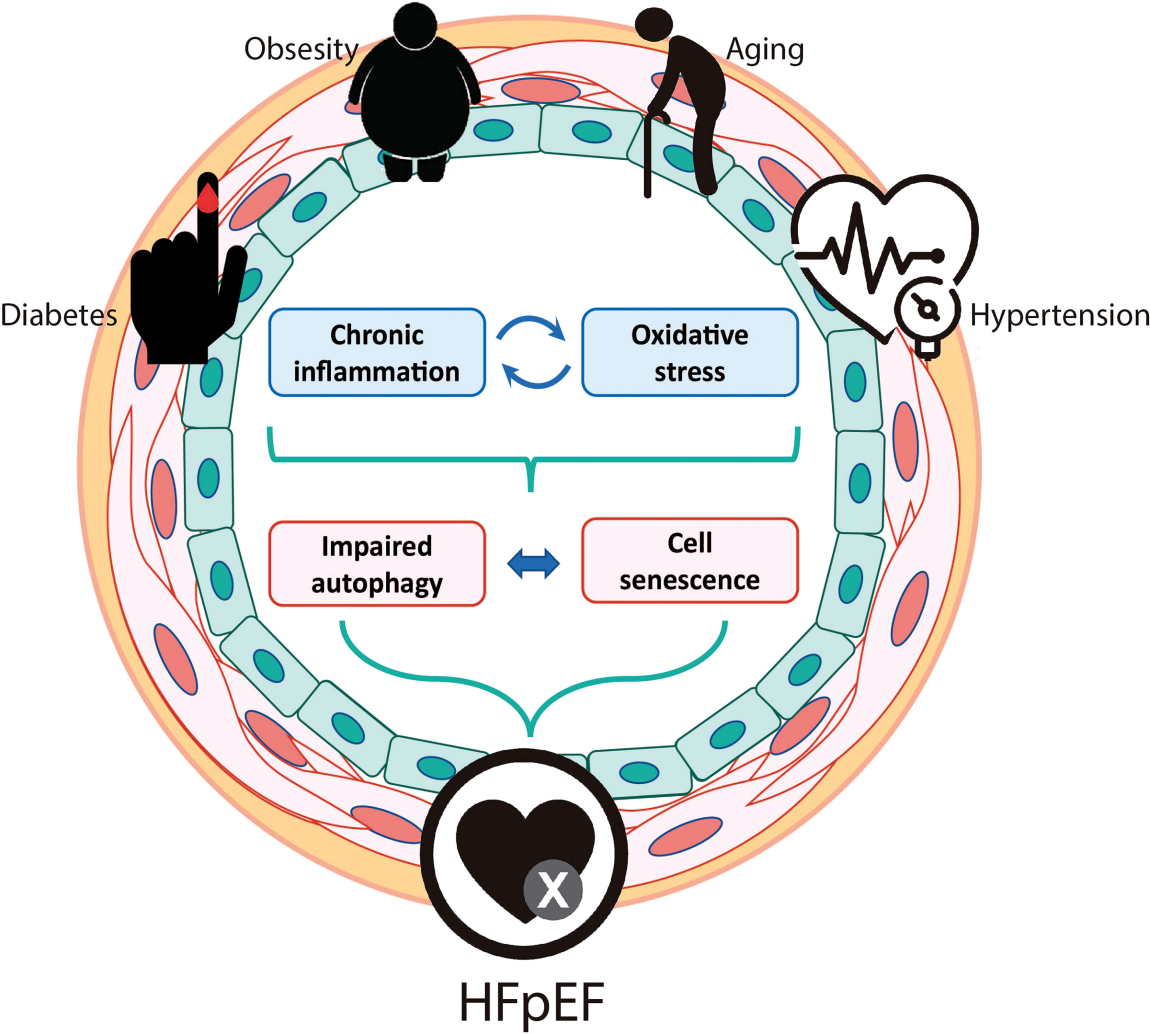
Potential role for vascular autophagy and senescence in the development and progression of HFpEF. Predisposing risk factors for HFpEF include older age, diabetes, obesity, and arterial hypertension. All these conditions trigger chronic inflammation and oxidative stress that could impair autophagy and induce cell senescence in both vascular smooth muscle cells and endothelial cells. Vascular senescence could be the responsible for the micro and macrovascular dysfunctions that are described in HFpEF patients and animal models.

In this review, we propose a potential role for vascular autophagy and senescence in the development and progression of HFpEF, focusing on both VSMCs and ECs. Diabetes, obesity, aging and hypertension, main risk factors for HFpEF, triggers chronic inflammation and ROS, that impairs autophagy and triggers cell senescence. The presented data support the idea that both autophagy and cell senescence, processes that are strongly related to one another, might be important components of HFpEF pathophysiology (**Figure 2**). As mentioned, HFpEF is an increasing healthcare burden worldwide, with no effective treatment. So, it is highly important to open new fields of research that could lead to a better understanding of this disease and the development of new therapeutic targets for its treatment.

## Author contributions

The authors confirm contribution to the paper as follows: review conception and design: FS-O, MT, FP, IN-S, and MC; critical analysis of the literature: FS-O, MT, FP, JM-B, IN-S, JR, MV, and MC; draft manuscript preparation: FS-O, MT, FP, JM-B, IN-S, JR, MV, SL, PC, and MC; SL and MC reviewed the final version of the manuscript. All authors approved the final version of the manuscript.

## Funding

The authors received funding from the Agencia Nacional de Investigación y Desarrollo (ANID), Chile: FONDAP 15130011, Fondecyt 1180157, Fondecyt 1220392, and Postdoctoral fellowship 3210496.

## Conflict of interest

The authors declare that the research was conducted in the absence of any commercial or financial relationships that could be construed as a potential conflict of interest.

## Publisher’s note

All claims expressed in this article are solely those of the authors and do not necessarily represent those of their affiliated organizations, or those of the publisher, the editors and the reviewers. Any product that may be evaluated in this article, or claim that may be made by its manufacturer, is not guaranteed or endorsed by the publisher.

## References

[1] WHO . Cardiovascular diseases (CVDs) (2022). Available at: https://www.who.int/en/news-room/fact-sheets/detail/cardiovascular-diseases-(cvds) (Accessed 09/21/2022).

[2] Tanai E Frantz S . Pathophysiology of heart failure. Compr Physiol (2015) 6:187–214. doi: 10.1002/cphy.c140055 26756631

[3] Dunlay SM Roger VL Redfield MM . Epidemiology of heart failure with preserved ejection fraction. Nat Rev Cardiol (2017) 14:591–602. doi: 10.1038/nrcardio.2017.65 28492288

[4] Heidenreich PA Albert NM Allen LA Bluemke DA Butler J Fonarow GC . Forecasting the impact of heart failure in the united states: a policy statement from the American heart association. Circ Heart Fail (2013) 6:606–19. doi: 10.1161/HHF.0b013e318291329a PMC3908895 23616602

[5] Redfield MM . Heart failure with preserved ejection fraction. N Engl J Med (2016) 375:1868–77. doi: 10.1056/NEJMcp1511175 27959663

[6] Simmonds SJ Cuijpers I Heymans S Jones E . Cellular and molecular differences between HFpEF and HFrEF: A step ahead in an improved pathological understanding. Cells (2020) 9:242. doi: 10.3390/cells9010242 31963679 PMC7016826

[7] Paulus WJ Tschope C . A novel paradigm for heart failure with preserved ejection fraction: comorbidities drive myocardial dysfunction and remodeling through coronary microvascular endothelial inflammation. J Am Coll Cardiol (2013) 62:263–71. doi: 10.1016/j.jacc.2013.02.092 23684677

[8] Larson KF Malik A Brozovich FV . Aging and heart failure with preserved ejection fraction. Compr Physiol (2022) 12:1–10. doi: 10.1002/cphy.c210035 35950652

[9] Gladden JD Linke WA Redfield MM . Heart failure with preserved ejection fraction. Pflugers Arch (2014) 466:1037–53. doi: 10.1007/s00424-014-1480-8 PMC4075067 24663384

[10] Boyle JP Thompson TJ Gregg EW Barker LE Williamson DF . Projection of the year 2050 burden of diabetes in the US adult population: dynamic modeling of incidence, mortality, and prediabetes prevalence. Popul Health Metr (2010) 8:29. doi: 10.1186/1478-7954-8-29 20969750 PMC2984379

[11] Finkelstein EA Khavjou OA Thompson H Trogdon JG Pan L Sherry B . Obesity and severe obesity forecasts through 2030. Am J Prev Med (2012) 42:563–70. doi: 10.1016/j.amepre.2011.10.026 22608371

[12] Ceia F Fonseca C Mota T Morais H Matias F De Sousa A . Prevalence of chronic heart failure in southwestern Europe: the EPICA study. Eur J Heart Fail (2002) 4:531–9. doi: 10.1016/s1388-9842(02)00034-x 12167394

[13] Ho JE Enserro D Brouwers FP Kizer JR Shah SJ Psaty BM . Predicting heart failure with preserved and reduced ejection fraction: The international collaboration on heart failure subtypes. Circ Heart Fail (2016) 9:e003116. doi: 10.1161/CIRCHEARTFAILURE.115.003116 27266854 PMC4902276

[14] Pieske B Tschope C De Boer RA Fraser AG Anker SD Donal E . How to diagnose heart failure with preserved ejection fraction: the HFA-PEFF diagnostic algorithm: a consensus recommendation from the heart failure association (HFA) of the European society of cardiology (ESC). Eur J Heart Fail (2020) 22:391–412. doi: 10.1002/ejhf.1741 32133741

[15] Lam CSP Voors AA De Boer RA Solomon SD Van Veldhuisen DJ . Heart failure with preserved ejection fraction: from mechanisms to therapies. Eur Heart J (2018) 39:2780–92. doi: 10.1093/eurheartj/ehy301 29905796

[16] Lee JF Barrett-O'keefe Z Garten RS Nelson AD Ryan JJ Nativi JN . Evidence of microvascular dysfunction in heart failure with preserved ejection fraction. Heart (2016) 102:278–84. doi: 10.1136/heartjnl-2015-308403 PMC4866903 26567228

[17] Lyle MA Brozovich FV . HFpEF, a disease of the vasculature: A closer look at the other half. Mayo Clin Proc (2018) 93:1305–14. doi: 10.1016/j.mayocp.2018.05.001 30064827

[18] Schiattarella GG Altamirano F Tong D French KM Villalobos E Kim SY . Nitrosative stress drives heart failure with preserved ejection fraction. Nature (2019) 568:351–6. doi: 10.1038/s41586-019-1100-z PMC6635957 30971818

[19] Yuan SY Rigor RR . Structure and function of exchange microvessels. In: Regulation of endothelial barrier function. San Rafael (CA: Morgan & Claypool Life Sciences (2010). 21634066

[20] Secomb TW Pries AR . The microcirculation: physiology at the mesoscale. J Physiol (2011) 589:1047–52. doi: 10.1113/jphysiol.2010.201541 PMC3060585 21242255

[21] Vancheri F Longo G Vancheri S Henein M . Coronary microvascular dysfunction. J Clin Med (2020) 9:2880. doi: 10.3390/jcm9092880 32899944 PMC7563453

[22] Horton WB Barrett EJ . Microvascular dysfunction in diabetes mellitus and cardiometabolic disease. Endocr Rev (2021) 42:29–55. doi: 10.1210/endrev/bnaa025 33125468 PMC7846151

[23] Kalogeropoulos A Georgiopoulou V Psaty BM Rodondi N Smith AL Harrison DG . Inflammatory markers and incident heart failure risk in older adults: the health ABC (Health, aging, and body composition) study. J Am Coll Cardiol (2010) 55:2129–37. doi: 10.1016/j.jacc.2009.12.045 PMC3267799 20447537

[24] Shear FE . Novel paradigms in the therapeutic management of heart failure with preserved ejection fraction: clinical perspectives. Am J Cardiovasc Dis (2019) 9:91–108. 31763061 PMC6872467

[25] Shah SJ Lam CSP Svedlund S Saraste A Hage C Tan RS . Prevalence and correlates of coronary microvascular dysfunction in heart failure with preserved ejection fraction: PROMIS-HFpEF. Eur Heart J (2018) 39:3439–50. doi: 10.1093/eurheartj/ehy531 PMC6927847 30165580

[26] Kitzman DW Nicklas B Kraus WE Lyles MF Eggebeen J Morgan TM . Skeletal muscle abnormalities and exercise intolerance in older patients with heart failure and preserved ejection fraction. Am J Physiol Heart Circ Physiol (2014) 306:H1364–1370. doi: 10.1152/ajpheart.00004.2014 PMC4010668 24658015

[27] Mohammed SF Hussain S Mirzoyev SA Edwards WD Maleszewski JJ Redfield MM . Coronary microvascular rarefaction and myocardial fibrosis in heart failure with preserved ejection fraction. Circulation (2015) 131:550–9. doi: 10.1161/CIRCULATIONAHA.114.009625 PMC4324362 25552356

[28] Yang JH Obokata M Reddy YNV Redfield MM Lerman A Borlaug BA . Endothelium-dependent and independent coronary microvascular dysfunction in patients with heart failure with preserved ejection fraction. Eur J Heart Fail (2020) 22:432–41. doi: 10.1002/ejhf.1671 31840366

[29] Weerts J Mourmans SGJ Barandiaran Aizpurua A Schroen BLM Knackstedt C Eringa E . The role of systemic microvascular dysfunction in heart failure with preserved ejection fraction. Biomolecules (2022) 12:278. doi: 10.3390/biom12020278 35204779 PMC8961612

[30] de Couto G Mesquita T Wu X Rajewski A Huang F Akhmerov A . Cell therapy attenuates endothelial dysfunction in hypertensive rats with heart failure and preserved ejection fraction. Am J Physiol Heart Circ Physiol (2022) 323:H892–903. doi: 10.1152/ajpheart.00287.2022 PMC9602891 36083797

[31] Franssen C Chen S Unger A Korkmaz HI De Keulenaer GW Tschöpe C . Myocardial Microvascular Inflammatory Endothelial Activation in Heart Failure With Preserved Ejection Fraction. JACC Heart Fail (2016) 4:312–24. doi: 10.1016/j.jchf.2015.10.007 26682792

[32] Sadowski J Targonski R Cyganski P Nowek P Starek-Stelmaszczyk M Zajac K . Remodeling of retinal arterioles and carotid arteries in heart failure development-a preliminary study. J Clin Med (2022) 11:3721. doi: 10.3390/jcm11133721 35807006 PMC9267807

[33] Chandra A Seidelmann SB Claggett BL Klein BE Klein R Shah AM . The association of retinal vessel calibres with heart failure and long-term alterations in cardiac structure and function: the atherosclerosis risk in communities (ARIC) study. Eur J Heart Fail (2019) 21:1207–15. doi: 10.1002/ejhf.1564 PMC12611558 31373139

[34] Yuksel S Yuksel EP Meric M . Abnormal nailfold videocapillaroscopic findings in heart failure patients with preserved ejection fraction. Clin Hemorheol Microcirc (2021) 77:115–21. doi: 10.3233/CH-200968 32925003

[35] Cecelja M Chowienczyk P . Role of arterial stiffness in cardiovascular disease. JRSM Cardiovasc Dis (2012) 1:cvd.2012.012016. doi: 10.1258/cvd.2012.012016 24175067 PMC3738327

[36] Hundley WG Kitzman DW Morgan TM Hamilton CA Darty SN Stewart KP . Cardiac cycle-dependent changes in aortic area and distensibility are reduced in older patients with isolated diastolic heart failure and correlate with exercise intolerance. J Am Coll Cardiol (2001) 38:796–802. doi: 10.1016/s0735-1097(01)01447-4 11527636

[37] Kishimoto S Kajikawa M Maruhashi T Iwamoto Y Matsumoto T Iwamoto A . Endothelial dysfunction and abnormal vascular structure are simultaneously present in patients with heart failure with preserved ejection fraction. Int J Cardiol (2017) 231:181–7. doi: 10.1016/j.ijcard.2017.01.024 28082090

[38] Borlaug BA Melenovsky V Russell SD Kessler K Pacak K Becker LC . Impaired chronotropic and vasodilator reserves limit exercise capacity in patients with heart failure and a preserved ejection fraction. Circulation (2006) 114:2138–47. doi: 10.1161/CIRCULATIONAHA.106.632745 17088459

[39] Borlaug BA Olson TP Lam CS Flood KS Lerman A Johnson BD . Global cardiovascular reserve dysfunction in heart failure with preserved ejection fraction. J Am Coll Cardiol (2010) 56:845–54. doi: 10.1016/j.jacc.2010.03.077 PMC2950645 20813282

[40] Kawaguchi M Hay I Fetics B Kass DA . Combined ventricular systolic and arterial stiffening in patients with heart failure and preserved ejection fraction: implications for systolic and diastolic reserve limitations. Circulation (2003) 107:714–20. doi: 10.1161/01.cir.0000048123.22359.a0 12578874

[41] Tartiere-Kesri L Tartiere JM Logeart D Beauvais F Cohen Solal A . Increased proximal arterial stiffness and cardiac response with moderate exercise in patients with heart failure and preserved ejection fraction. J Am Coll Cardiol (2012) 59:455–61. doi: 10.1016/j.jacc.2011.10.873 22281248

[42] Reddy YNV Andersen MJ Obokata M Koepp KE Kane GC Melenovsky V . Arterial stiffening with exercise in patients with heart failure and preserved ejection fraction. J Am Coll Cardiol (2017) 70:136–48. doi: 10.1016/j.jacc.2017.05.029 PMC5520668 28683960

[43] Schwartzenberg S Redfield MM From AM Sorajja P Nishimura RA Borlaug BA . Effects of vasodilation in heart failure with preserved or reduced ejection fraction implications of distinct pathophysiologies on response to therapy. J Am Coll Cardiol (2012) 59:442–51. doi: 10.1016/j.jacc.2011.09.062 22281246

[44] Fernandes VR Polak JF Cheng S Rosen BD Carvalho B Nasir K . Arterial stiffness is associated with regional ventricular systolic and diastolic dysfunction: the multi-ethnic study of atherosclerosis. Arterioscler Thromb Vasc Biol (2008) 28:194–201. doi: 10.1161/ATVBAHA.107.156950 17962621

[45] Hu Y Li L Shen L Gao H . The relationship between arterial wall stiffness and left ventricular dysfunction. Neth Heart J (2013) 21:222–7. doi: 10.1007/s12471-012-0353-z PMC3636336 23203731

[46] Aladin AI Soliman EZ Kitzman DW Dardari Z Rasool SH Yeboah J . Comparison of the relation of carotid intima-media thickness with incident heart failure with reduced versus preserved ejection fraction (from the multi-ethnic study of atherosclerosis [MESA]). Am J Cardiol (2021) 148:102–9. doi: 10.1016/j.amjcard.2021.02.020 PMC8113133 33667446

[47] Puato M Boschetti G Rattazzi M Zanon M Pesavento R Faggin E . Intima-media thickness remodelling in hypertensive subjects with long-term well-controlled blood pressure levels. Blood Press (2017) 26:48–53. doi: 10.1080/08037051.2016.1184964 27216375

[48] Engstrom G Melander O Hedblad B . Carotid intima-media thickness, systemic inflammation, and incidence of heart failure hospitalizations. Arterioscler Thromb Vasc Biol (2009) 29:1691–5. doi: 10.1161/ATVBAHA.109.193490 19644052

[49] Hu W Zhang H Liu Z Duan Q Liu J Dong Q . Relationship between adipose tissue distribution and arterial stiffness in HFpEF. Nutrition (2022) 102:111726. doi: 10.1016/j.nut.2022.111726 35843103

[50] Hundley WG Bayram E Hamilton CA Hamilton EA Morgan TM Darty SN . Leg flow-mediated arterial dilation in elderly patients with heart failure and normal left ventricular ejection fraction. Am J Physiol Heart Circ Physiol (2007) 292:H1427–1434. doi: 10.1152/ajpheart.00567.2006 17085542

[51] Farrero M Blanco I Batlle M Santiago E Cardona M Vidal B . Pulmonary hypertension is related to peripheral endothelial dysfunction in heart failure with preserved ejection fraction. Circ Heart Fail (2014) 7:791–8. doi: 10.1161/CIRCHEARTFAILURE.113.000942 25047042

[52] Bayes-Genis A Cediel G Domingo M Codina P Santiago E Lupon J . Biomarkers in heart failure with preserved ejection fraction. Card Fail Rev (2022) 8:e20. doi: 10.15420/cfr.2021.37 35815256 PMC9253965

[53] Katsuumi G Shimizu I Yoshida Y Minamino T . Vascular senescence in cardiovascular and metabolic diseases. Front Cardiovasc Med (2018) 5:18. doi: 10.3389/fcvm.2018.00018 29556500 PMC5845435

[54] Lopez-Otin C Blasco MA Partridge L Serrano M Kroemer G . The hallmarks of aging. Cell (2013) 153:1194–217. doi: 10.1016/j.cell.2013.05.039 PMC3836174 23746838

[55] Hernandez-Segura A Nehme J Demaria M . Hallmarks of cellular senescence. Trends Cell Biol (2018) 28:436–53. doi: 10.1016/j.tcb.2018.02.001 29477613

[56] Birch J Gil J . Senescence and the SASP: many therapeutic avenues. Genes Dev (2020) 34:1565–76. doi: 10.1101/gad.343129.120 PMC7706700 33262144

[57] Dimri GP Lee X Basile G Acosta M Scott G Roskelley C . A biomarker that identifies senescent human cells in culture and in aging skin *in vivo* . Proc Natl Acad Sci U.S.A. (1995) 92:9363–7. doi: 10.1073/pnas.92.20.9363 PMC40985 7568133

[58] Iwasa H Han J Ishikawa F . Mitogen-activated protein kinase p38 defines the common senescence-signalling pathway. Genes Cells (2003) 8:131–44. doi: 10.1046/j.1365-2443.2003.00620.x 12581156

[59] Minamino T Komuro I . Vascular cell senescence: contribution to atherosclerosis. Circ Res (2007) 100:15–26. doi: 10.1161/01.RES.0000256837.40544.4a 17204661

[60] Munoz-Espin D Serrano M . Cellular senescence: from physiology to pathology. Nat Rev Mol Cell Biol (2014) 15:482–96. doi: 10.1038/nrm3823 24954210

[61] Salama R Sadaie M Hoare M Narita M . Cellular senescence and its effector programs. Genes Dev (2014) 28:99–114. doi: 10.1101/gad.235184.113 24449267 PMC3909793

[62] van Deursen JM . The role of senescent cells in ageing. Nature (2014) 509:439–46. doi: 10.1038/nature13193 PMC4214092 24848057

[63] El Assar M Angulo J Vallejo S Peiro C Sanchez-Ferrer CF Rodriguez-Manas L . Mechanisms involved in the aging-induced vascular dysfunction. Front Physiol (2012) 3:132. doi: 10.3389/fphys.2012.00132 22783194 PMC3361078

[64] Jia G Aroor AR Jia C Sowers JR . Endothelial cell senescence in aging-related vascular dysfunction. Biochim Biophys Acta Mol Basis Dis (2019) 1865:1802–9. doi: 10.1016/j.bbadis.2018.08.008 31109450

[65] Kato H Maezawa Y . Atherosclerosis and cardiovascular diseases in progeroid syndromes. J Atheroscler Thromb (2022) 29:439–47. doi: 10.5551/jat.RV17061 PMC9100459 34511576

[66] Lakatta EG Levy D . Arterial and cardiac aging: major shareholders in cardiovascular disease enterprises: Part I: aging arteries: a "set up" for vascular disease. Circulation (2003) 107:139–46. doi: 10.1161/01.cir.0000048892.83521.58 12515756

[67] Lakatta EG . The reality of aging viewed from the arterial wall. Artery Res (2013) 7:73–80. doi: 10.1016/j.artres.2013.01.003 23667404 PMC3646655

[68] Spina M Garbisa S Hinnie J Hunter JC Serafini-Fracassini A . Age-related changes in composition and mechanical properties of the tunica media of the upper thoracic human aorta. Arteriosclerosis (1983) 3:64–76. doi: 10.1161/01.atv.3.1.64 6824497

[69] Gerstenblith G Frederiksen J Yin FC Fortuin NJ Lakatta EG Weisfeldt ML . Echocardiographic assessment of a normal adult aging population. Circulation (1977) 56:273–8. doi: 10.1161/01.cir.56.2.273 872321

[70] Mauriello A Orlandi A Palmieri G Spagnoli LG Oberholzer M Christen H . Age-related modification of average volume and anisotropy of vascular smooth muscle cells. Pathol Res Pract (1992) 188:630–6. doi: 10.1016/S0344-0338(11)80070-1 1409102

[71] Harvey A Montezano AC Touyz RM . Vascular biology of ageing-implications in hypertension. J Mol Cell Cardiol (2015) 83:112–21. doi: 10.1016/j.yjmcc.2015.04.011 PMC4534766 25896391

[72] Minamino T Miyauchi H Yoshida T Ishida Y Yoshida H Komuro I . Endothelial cell senescence in human atherosclerosis: role of telomere in endothelial dysfunction. Circulation (2002) 105:1541–4. doi: 10.1161/01.cir.0000013836.85741.17 11927518

[73] Marchand A Atassi F Gaaya A Leprince P Le Feuvre C Soubrier F . The wnt/beta-catenin pathway is activated during advanced arterial aging in humans. Aging Cell (2011) 10:220–32. doi: 10.1111/j.1474-9726.2010.00661.x 21108734

[74] Cafueri G Parodi F Pistorio A Bertolotto M Ventura F Gambini C . Endothelial and smooth muscle cells from abdominal aortic aneurysm have increased oxidative stress and telomere attrition. PloS One (2012) 7:e35312. doi: 10.1371/journal.pone.0035312 22514726 PMC3325957

[75] Morgan RG Ives SJ Lesniewski LA Cawthon RM Andtbacka RH Noyes RD . Age-related telomere uncapping is associated with cellular senescence and inflammation independent of telomere shortening in human arteries. Am J Physiol Heart Circ Physiol (2013) 305:H251–258. doi: 10.1152/ajpheart.00197.2013 PMC3726958 23666675

[76] Melk A Schmidt BM Takeuchi O Sawitzki B Rayner DC Halloran PF . Expression of p16INK4a and other cell cycle regulator and senescence associated genes in aging human kidney. Kidney Int (2004) 65:510–20. doi: 10.1111/j.1523-1755.2004.00438.x 14717921

[77] Yang D Mccrann DJ Nguyen H St Hilaire C Depinho RA Jones MR . Increased polyploidy in aortic vascular smooth muscle cells during aging is marked by cellular senescence. Aging Cell (2007) 6:257–60. doi: 10.1111/j.1474-9726.2007.00274.x PMC3303600 17291294

[78] Rajapakse AG Yepuri G Carvas JM Stein S Matter CM Scerri I . Hyperactive S6K1 mediates oxidative stress and endothelial dysfunction in aging: inhibition by resveratrol. PloS One (2011) 6:e19237. doi: 10.1371/journal.pone.0019237 21544240 PMC3081344

[79] Lin JR Shen WL Yan C Gao PJ . Downregulation of dynamin-related protein 1 contributes to impaired autophagic flux and angiogenic function in senescent endothelial cells. Arterioscler Thromb Vasc Biol (2015) 35:1413–22. doi: 10.1161/ATVBAHA.115.305706 25908761

[80] Simoes G Pereira T Caseiro A . Matrix metaloproteinases in vascular pathology. Microvasc Res (2022) 143:104398. doi: 10.1016/j.mvr.2022.104398 35671836

[81] Lacolley P Regnault V Nicoletti A Li Z Michel JB . The vascular smooth muscle cell in arterial pathology: a cell that can take on multiple roles. Cardiovasc Res (2012) 95:194–204. doi: 10.1093/cvr/cvs135 22467316

[82] Faggiotto A Ross R Harker L . Studies of hypercholesterolemia in the nonhuman primate. i. changes that lead to fatty streak formation. Arteriosclerosis (1984) 4:323–40. doi: 10.1161/01.atv.4.4.323 6466191

[83] Lakatta EG . So! what's aging? is cardiovascular aging a disease? J Mol Cell Cardiol (2015) 83:1–13. doi: 10.1016/j.yjmcc.2015.04.005 25870157 PMC4532266

[84] Shi J Yang Y Cheng A Xu G He F . Metabolism of vascular smooth muscle cells in vascular diseases. Am J Physiol Heart Circ Physiol (2020) 319:H613–31. doi: 10.1152/ajpheart.00220.2020 32762559

[85] Gomez D Owens GK . Smooth muscle cell phenotypic switching in atherosclerosis. Cardiovasc Res (2012) 95:156–64. doi: 10.1093/cvr/cvs115 PMC3388816 22406749

[86] Matthews C Gorenne I Scott S Figg N Kirkpatrick P Ritchie A . Vascular smooth muscle cells undergo telomere-based senescence in human atherosclerosis: effects of telomerase and oxidative stress. Circ Res (2006) 99:156–64. doi: 10.1161/01.RES.0000233315.38086.bc 16794190

[87] Wang J Uryga AK Reinhold J Figg N Baker L Finigan A . Vascular smooth muscle cell senescence promotes atherosclerosis and features of plaque vulnerability. Circulation (2015) 132:1909–19. doi: 10.1161/CIRCULATIONAHA.115.016457 26416809

[88] Morgan RG Ives SJ Walker AE Cawthon RM Andtbacka RH Noyes D . Role of arterial telomere dysfunction in hypertension: relative contributions of telomere shortening and telomere uncapping. J Hypertens (2014) 32:1293–9. doi: 10.1097/HJH.0000000000000157 PMC4198301 24686009

[89] Durik M Kavousi M van der Pluijm I Isaacs A Cheng C Verdonk K . Nucleotide excision DNA repair is associated with age-related vascular dysfunction. Circulation (2012) 126:468–78. doi: 10.1161/CIRCULATIONAHA.112.104380 PMC3430727 22705887

[90] Westhoff JH Hilgers KF Steinbach MP Hartner A Klanke B Amann K . Hypertension induces somatic cellular senescence in rats and humans by induction of cell cycle inhibitor p16INK4a. Hypertension (2008) 52:123–9. doi: 10.1161/HYPERTENSIONAHA.107.099432 18504326

[91] Gardner SE Humphry M Bennett MR Clarke MC . Senescent vascular smooth muscle cells drive inflammation through an interleukin-1alpha-Dependent senescence-associated secretory phenotype. Arterioscler Thromb Vasc Biol (2015) 35:1963–74. doi: 10.1161/ATVBAHA.115.305896 PMC4548545 26139463

[92] Herbert KE Mistry Y Hastings R Poolman T Niklason L Williams B . Angiotensin II-mediated oxidative DNA damage accelerates cellular senescence in cultured human vascular smooth muscle cells *via* telomere-dependent and independent pathways. Circ Res (2008) 102:201–8. doi: 10.1161/CIRCRESAHA.107.158626 PMC2861985 17991883

[93] Zhao W Zheng XL Peng DQ Zhao SP . Myocyte enhancer factor 2A regulates hydrogen peroxide-induced senescence of vascular smooth muscle cells *Via* microRNA-143. J Cell Physiol (2015) 230:2202–11. doi: 10.1002/jcp.24948 25655189

[94] Kunieda T Minamino T Nishi J Tateno K Oyama T Katsuno T . Angiotensin II induces premature senescence of vascular smooth muscle cells and accelerates the development of atherosclerosis *via* a p21-dependent pathway. Circulation (2006) 114:953–60. doi: 10.1161/CIRCULATIONAHA.106.626606 16908765

[95] Miao SB Xie XL Yin YJ Zhao LL Zhang F Shu YN . Accumulation of smooth muscle 22alpha protein accelerates senescence of vascular smooth muscle cells *via* stabilization of p53 *In vitro* and *In vivo* . Arterioscler Thromb Vasc Biol (2017) 37:1849–59. doi: 10.1161/ATVBAHA.117.309378 28798142

[96] Mondaca-Ruff D Riquelme JA Quiroga C Norambuena-Soto I Sanhueza-Olivares F Villar-Fincheira P . Angiotensin II-regulated autophagy is required for vascular smooth muscle cell hypertrophy. Front Pharmacol (2018) 9:1553. doi: 10.3389/fphar.2018.01553 30804791 PMC6371839

[97] Minamino T Mitsialis SA Kourembanas S . Hypoxia extends the life span of vascular smooth muscle cells through telomerase activation. Mol Cell Biol (2001) 21:3336–42. doi: 10.1128/MCB.21.10.3336-3342.2001 PMC100255 11313459

[98] Chi C Li DJ Jiang YJ Tong J Fu H Wu YH . Vascular smooth muscle cell senescence and age-related diseases: State of the art. Biochim Biophys Acta Mol Basis Dis (2019) 1865:1810–21. doi: 10.1016/j.bbadis.2018.08.015 31109451

[99] Glick D Barth S Macleod KF . Autophagy: cellular and molecular mechanisms. J Pathol (2010) 221:3–12. doi: 10.1002/path.2697 20225336 PMC2990190

[100] De Meyer GR Grootaert MO Michiels CF Kurdi A Schrijvers DM Martinet W . Autophagy in vascular disease. Circ Res (2015) 116:468–79. doi: 10.1161/CIRCRESAHA.116.303804 25634970

[101] Luo Z Xu W Ma S Qiao H Gao L Zhang R . Moderate autophagy inhibits vascular smooth muscle cell senescence to stabilize progressed atherosclerotic plaque *via* the mTORC1/ULK1/ATG13 signal pathway. Oxid Med Cell Longev (2017) 2017:3018190. doi: 10.1155/2017/3018190 28713484 PMC5497616

[102] Yin H Pickering JG . Cellular senescence and vascular disease: Novel routes to better understanding and therapy. Can J Cardiol (2016) 32:612–23. doi: 10.1016/j.cjca.2016.02.051 27040096

[103] Stojanovic SD Fuchs M Kunz M Xiao K Just A Pich A . Inflammatory drivers of cardiovascular disease: Molecular characterization of senescent coronary vascular smooth muscle cells. Front Physiol (2020) 11:520. doi: 10.3389/fphys.2020.00520 32523550 PMC7261939

[104] Alexander Y Osto E Schmidt-Trucksass A Shechter M Trifunovic D Duncker DJ . Endothelial function in cardiovascular medicine: a consensus paper of the European society of cardiology working groups on atherosclerosis and vascular biology, aorta and peripheral vascular diseases, coronary pathophysiology and microcirculation, and thrombosis. Cardiovasc Res (2021) 117:29–42. doi: 10.1093/cvr/cvaa085 32282914 PMC7797212

[105] Neubauer K Zieger B . Endothelial cells and coagulation. Cell Tissue Res (2022) 387:391–8. doi: 10.1007/s00441-021-03471-2 PMC8975780 34014399

[106] Bloom SI Islam MT Lesniewski LA Donato AJ . Mechanisms and consequences of endothelial cell senescence. Nat Rev Cardiol (2022). doi: 10.1038/s41569-022-00739-0 PMC10026597 35853997

[107] Burns EM Kruckeberg TW Comerford LE Buschmann MT . Thinning of capillary walls and declining numbers of endothelial mitochondria in the cerebral cortex of the aging primate, macaca nemestrina. J Gerontol (1979) 34:642–50. doi: 10.1093/geronj/34.5.642 112144

[108] Ungvari Z Orosz Z Labinskyy N Rivera A Xiangmin Z Smith K . Increased mitochondrial H2O2 production promotes endothelial NF-kappaB activation in aged rat arteries. Am J Physiol Heart Circ Physiol (2007) 293:H37–47. doi: 10.1152/ajpheart.01346.2006 17416599

[109] Toth P Tarantini S Springo Z Tucsek Z Gautam T Giles CB . Aging exacerbates hypertension-induced cerebral microhemorrhages in mice: role of resveratrol treatment in vasoprotection. Aging Cell (2015) 14:400–8. doi: 10.1111/acel.12315 PMC4406669 25677910

[110] Donato AJ Magerko KA Lawson BR Durrant JR Lesniewski LA Seals DR . SIRT-1 and vascular endothelial dysfunction with ageing in mice and humans. J Physiol (2011) 589:4545–54. doi: 10.1113/jphysiol.2011.211219 PMC3208223 21746786

[111] Liu H Chen L Xiao W Liu J Long C Zhan W . Alteration of E2F2 expression in governing endothelial cell senescence. Genes (Basel) (2022) 13:1522. doi: 10.3390/genes13091522 36140689 PMC9498592

[112] Kida Y Goligorsky MS . Sirtuins, cell senescence, and vascular aging. Can J Cardiol (2016) 32:634–41. doi: 10.1016/j.cjca.2015.11.022 PMC4848124 26948035

[113] Conti V Corbi G Polito MV Ciccarelli M Manzo V Torsiello M . Sirt1 activity in PBMCs as a biomarker of different heart failure phenotypes. Biomolecules (2020) 10:1590. doi: 10.3390/biom10111590 33238655 PMC7700185

[114] Kohn JC Lampi MC Reinhart-King CA . Age-related vascular stiffening: causes and consequences. Front Genet (2015) 6:112. doi: 10.3389/fgene.2015.00112 25926844 PMC4396535

[115] Fleenor BS Marshall KD Rippe C Seals DR . Replicative aging induces endothelial to mesenchymal transition in human aortic endothelial cells: potential role of inflammation. J Vasc Res (2012) 49:59–64. doi: 10.1159/000329681 21985896 PMC3214888

[116] Schlatmann TJ Becker AE . Histologic changes in the normal aging aorta: implications for dissecting aortic aneurysm. Am J Cardiol (1977) 39:13–20. doi: 10.1016/s0002-9149(77)80004-0 831420

[117] Greenberg SR . The association of medial collagenous tissue with atheroma formation in the aging human aorta as revealed by a special technique. Histol Histopathol (1986) 1:323–6. 2980126

[118] Fleenor BS Marshall KD Durrant JR Lesniewski LA Seals DR . Arterial stiffening with ageing is associated with transforming growth factor-beta1-related changes in adventitial collagen: reversal by aerobic exercise. J Physiol (2010) 588:3971–82. doi: 10.1113/jphysiol.2010.194753 PMC3000586 20807791

[119] Bonnema DD Webb CS Pennington WR Stroud RE Leonardi AE Clark LL . Effects of age on plasma matrix metalloproteinases (MMPs) and tissue inhibitor of metalloproteinases (TIMPs). J Card Fail (2007) 13:530–40. doi: 10.1016/j.cardfail.2007.04.010 PMC2698433 17826643

[120] McNulty M Spiers P Mcgovern E Feely J . Aging is associated with increased matrix metalloproteinase-2 activity in the human aorta. Am J Hypertens (2005) 18:504–9. doi: 10.1016/j.amjhyper.2004.11.011 15831360

[121] Puspitasari YM Diaz-Canestro C Sudano I Flammer A Bonetti NR Wuest P . The role of matrix metalloproteinase-2 on age-dependent arterial stiffness. Eur Heart J (2020) 41:3778. doi: 10.1093/ehjci/ehaa946.3778 33126262

[122] Medley TL Kingwell BA Gatzka CD Pillay P Cole TJ . Matrix metalloproteinase-3 genotype contributes to age-related aortic stiffening through modulation of gene and protein expression. Circ Res (2003) 92:1254–61. doi: 10.1161/01.RES.0000076891.24317.CA 12750310

[123] Ungvari Z Tarantini S Donato AJ Galvan V Csiszar A . Mechanisms of vascular aging. Circ Res (2018) 123:849–67. doi: 10.1161/CIRCRESAHA.118.311378 PMC6248882 30355080

[124] Wang M Kim SH Monticone RE Lakatta EG . Matrix metalloproteinases promote arterial remodeling in aging, hypertension, and atherosclerosis. Hypertension (2015) 65:698–703. doi: 10.1161/HYPERTENSIONAHA.114.03618 25667214 PMC4359070

[125] Summer G Kuhn AR Munts C Miranda-Silva D Leite-Moreira AF Lourenco AP . A directed network analysis of the cardiome identifies molecular pathways contributing to the development of HFpEF. J Mol Cell Cardiol (2020) 144:66–75. doi: 10.1016/j.yjmcc.2020.05.008 32422321

[126] Kobusiak-Prokopowicz M Krzysztofik J Kaaz K Jolda-Mydlowska B Mysiak A . MMP-2 and TIMP-2 in patients with heart failure and chronic kidney disease. Open Med (Wars) (2018) 13:237–46. doi: 10.1515/med-2018-0037 PMC6004519 29915813

[127] Goncalves PR Nascimento LD Gerlach RF Rodrigues KE Prado AF . Matrix metalloproteinase 2 as a pharmacological target in heart failure. Pharm (Basel) (2022) 15:920. doi: 10.3390/ph15080920 PMC9331741 35893744

[128] Zhang Y Van Laer AO Baicu CF Neff LS Hoffman S Katz MR . Phenotypic characterization of primary cardiac fibroblasts from patients with HFpEF. PloS One (2022) 17:e0262479. doi: 10.1371/journal.pone.0262479 35015787 PMC8752005

[129] Aman Y Schmauck-Medina T Hansen M Morimoto RI Simon AK Bjedov I . Autophagy in healthy aging and disease. Nat Aging (2021) 1:634–50. doi: 10.1038/s43587-021-00098-4 PMC8659158 34901876

[130] Grootaert MOJ Moulis M Roth L Martinet W Vindis C Bennett MR . Vascular smooth muscle cell death, autophagy and senescence in atherosclerosis. Cardiovasc Res (2018) 114:622–34. doi: 10.1093/cvr/cvy007 29360955

[131] Klionsky DJ Abeliovich H Agostinis P Agrawal DK Aliev G Askew DS . Guidelines for the use and interpretation of assays for monitoring autophagy in higher eukaryotes. Autophagy (2008) 4:151–75. doi: 10.4161/auto.5338 PMC2654259 18188003

[132] Lu H Du W Ren L Hamblin MH Becker RC Chen YE . Vascular smooth muscle cells in aortic aneurysm: From genetics to mechanisms. J Am Heart Assoc (2021) 10:e023601. doi: 10.1161/JAHA.121.023601 34796717 PMC9075263

[133] De Munck DG Leloup AJA De Meyer GRY Martinet W Fransen P . Defective autophagy in vascular smooth muscle cells increases passive stiffness of the mouse aortic vessel wall. Pflugers Arch (2020) 472:1031–40. doi: 10.1007/s00424-020-02408-y 32488322

[134] Grootaert MO Da Costa Martins PA Bitsch N Pintelon I De Meyer GR Martinet W . Defective autophagy in vascular smooth muscle cells accelerates senescence and promotes neointima formation and atherogenesis. Autophagy (2015) 11:2014–32. doi: 10.1080/15548627.2015.1096485 PMC4824610 26391655

[135] Frudd K Burgoyne T Burgoyne JR . Oxidation of Atg3 and Atg7 mediates inhibition of autophagy. Nat Commun (2018) 9:95. doi: 10.1038/s41467-017-02352-z 29311554 PMC5758830

[136] Sung JY Lee KY Kim JR Choi HC . Interaction between mTOR pathway inhibition and autophagy induction attenuates adriamycin-induced vascular smooth muscle cell senescence through decreased expressions of p53/p21/p16. Exp Gerontol (2018) 109:51–8. doi: 10.1016/j.exger.2017.08.001 28797827

[137] Tyrrell DJ Blin MG Song J Wood SC Zhang M Beard DA . Age-associated mitochondrial dysfunction accelerates atherogenesis. Circ Res (2020) 126:298–314. doi: 10.1161/CIRCRESAHA.119.315644 31818196 PMC7006722

[138] Hsieh PN Zhou G Yuan Y Zhang R Prosdocimo DA Sangwung P . A conserved KLF-autophagy pathway modulates nematode lifespan and mammalian age-associated vascular dysfunction. Nat Commun (2017) 8:914. doi: 10.1038/s41467-017-00899-5 29030550 PMC5640649

[139] Salazar G Cullen A Huang J Zhao Y Serino A Hilenski L . SQSTM1/p62 and PPARGC1A/PGC-1alpha at the interface of autophagy and vascular senescence. Autophagy (2020) 16:1092–110. doi: 10.1080/15548627.2019.1659612 PMC7469683 31441382

[140] Xu XJ Zhao WB Feng SB Sun C Chen Q Ni B . Celastrol alleviates angiotensin IImediated vascular smooth muscle cell senescence *via* induction of autophagy. Mol Med Rep (2017) 16:7657–64. doi: 10.3892/mmr.2017.7533 28944849

[141] Lee KY Kim JR Choi HC . Genistein-induced LKB1-AMPK activation inhibits senescence of VSMC through autophagy induction. Vascul Pharmacol (2016) 81:75–82. doi: 10.1016/j.vph.2016.02.007 26924458

[142] Kim SG Sung JY Kim JR Choi HC . Nifedipine-induced AMPK activation alleviates senescence by increasing autophagy and suppressing of Ca(2+) levels in vascular smooth muscle cells. Mech Ageing Dev (2020) 190:111314. doi: 10.1016/j.mad.2020.111314 32679123

[143] Bai HY Li H Zhou X Gu HB Shan BS . AT2 receptor stimulation inhibits vascular smooth muscle cell senescence induced by angiotensin II and hyperglycemia. Am J Hypertens (2022) 35:884–91. doi: 10.1093/ajh/hpac083 35793143

[144] Xu H Yu M Yu Y Li Y Yang F Liu Y . KLF4 prevented angiotensin II-induced smooth muscle cell senescence by enhancing autophagic activity. Eur J Clin Invest (2022) 52:e13804. doi: 10.1111/eci.13804 35506324

[145] Tan P Wang YJ Li S Wang Y He JY Chen YY . The PI3K/Akt/mTOR pathway regulates the replicative senescence of human VSMCs. Mol Cell Biochem (2016) 422:1–10. doi: 10.1007/s11010-016-2796-9 27619662

[146] Tan P Wang H Zhan J Ma X Cui X Wang Y . Rapamycininduced miR30a downregulation inhibits senescence of VSMCs by targeting Beclin1. Int J Mol Med (2019) 43:1311–20. doi: 10.3892/ijmm.2019.4074 PMC6365076 30747228

[147] Mameli E Martello A Caporali A . Autophagy at the interface of endothelial cell homeostasis and vascular disease. FEBS J (2022) 289:2976–91. doi: 10.1111/febs.15873 33934518

[148] Gogiraju R Hubert A Fahrer J Straub BK Brandt M Wenzel P . Endothelial leptin receptor deletion promotes cardiac autophagy and angiogenesis following pressure overload by suppressing Akt/mTOR signaling. Circ Heart Fail (2019) 12:e005622. doi: 10.1161/CIRCHEARTFAILURE.118.005622 30621510

[149] Luu AZ Luu VZ Chowdhury B Kosmopoulos A Pan Y Al-Omran M . Loss of endothelial cell-specific autophagy-related protein 7 exacerbates doxorubicin-induced cardiotoxicity. Biochem Biophys Rep (2021) 25:100926. doi: 10.1016/j.bbrep.2021.100926 33553688 PMC7851775

[150] Lin L Zhang L Li XT Ji JK Chen XQ Li YL . Rhynchophylline attenuates senescence of endothelial progenitor cells by enhancing autophagy. Front Pharmacol (2019) 10:1617. doi: 10.3389/fphar.2019.01617 32047439 PMC6997466

[151] Lee J Yoo JH Kim HS Cho YK Lee Y Lee WJ . C1q/TNF-related protein-9 attenuates palmitic acid-induced endothelial cell senescence *via* increasing autophagy. Mol Cell Endocrinol (2021) 521:111114. doi: 10.1016/j.mce.2020.111114 33301838

[152] Pan X Wu B Fan X Xu G Ou C Chen M . YAP accelerates vascular senescence *via* blocking autophagic flux and activating mTOR. J Cell Mol Med (2021) 25:170–83. doi: 10.1111/jcmm.15902 PMC7810949 33314583

[153] Ou H Huang Z Mo Z Xiao J . The characteristics and roles of advanced oxidation protein products in atherosclerosis. Cardiovasc Toxicol (2017) 17:1–12. doi: 10.1007/s12012-016-9377-8 27350146

[154] Chen Y Liu Z Chen H Huang X Huang X Lei Y . p53 SUMOylation mediates AOPP-induced endothelial senescence and apoptosis evasion. Front Cardiovasc Med (2021) 8:795747. doi: 10.3389/fcvm.2021.795747 35187108 PMC8850781

[155] Chen F Chen B Xiao FQ Wu YT Wang RH Sun ZW . Autophagy protects against senescence and apoptosis *via* the RAS-mitochondria in high-glucose-induced endothelial cells. Cell Physiol Biochem (2014) 33:1058–74. doi: 10.1159/000358676 24732710

[156] Gatica D Chiong M Lavandero S Klionsky DJ . The role of autophagy in cardiovascular pathology. Cardiovasc Res (2022) 118:934–50. doi: 10.1093/cvr/cvab158 PMC8930074 33956077

[157] Hahn VS Knutsdottir H Luo X Bedi K Margulies KB Haldar SM . Myocardial gene expression signatures in human heart failure with preserved ejection fraction. Circulation (2021) 143:120–34. doi: 10.1161/CIRCULATIONAHA.120.050498 PMC7856095 33118835

[158] Roh JD Houstis N Yu A Chang B Yeri A Li H . Exercise training reverses cardiac aging phenotypes associated with heart failure with preserved ejection fraction in male mice. Aging Cell (2020) 19:e13159. doi: 10.1111/acel.13159 32441410 PMC7294786

[159] Shinmura K Tamaki K Sano M Murata M Yamakawa H Ishida H . Impact of long-term caloric restriction on cardiac senescence: caloric restriction ameliorates cardiac diastolic dysfunction associated with aging. J Mol Cell Cardiol (2011) 50:117–27. doi: 10.1016/j.yjmcc.2010.10.018 20977912

[160] Hoshino A Mita Y Okawa Y Ariyoshi M Iwai-Kanai E Ueyama T . Cytosolic p53 inhibits parkin-mediated mitophagy and promotes mitochondrial dysfunction in the mouse heart. Nat Commun (2013) 4:2308. doi: 10.1038/ncomms3308 23917356

[161] Yang H-J Kong B Shuai W Zhang JJ Huang H . MD1 deletion exaggerates cardiomyocyte autophagy induced by heart failure with preserved ejection fraction through ROS/MAPK signalling pathway. J Cell Mol Med (2020) 24:9300–12. doi: 10.1111/jcmm.15579 PMC7417689 32648659

[162] Brown DA Perry JB Allen ME Sabbah HN Stauffer BL Shaikh SR . Expert consensus document: Mitochondrial function as a therapeutic target in heart failure. Nat Rev Cardiol (2017) 14:238–50. doi: 10.1038/nrcardio.2016.203 PMC5350035 28004807

[163] Gevaert AB Shakeri H Leloup AJ Van Hove CE De Meyer GRY Vrints CJ . Endothelial senescence contributes to heart failure with preserved ejection fraction in an aging mouse model. Circ Heart Fail (2017) 10:e003806. doi: 10.1161/CIRCHEARTFAILURE.116.003806 28611124

[164] Van Linthout S Tschope C . Inflammation - cause or consequence of heart failure or both? Curr Heart Fail Rep (2017) 14:251–65. doi: 10.1007/s11897-017-0337-9 PMC5527060 28667492

[165] Frieler RA Mortensen RM . Immune cell and other noncardiomyocyte regulation of cardiac hypertrophy and remodeling. Circulation (2015) 131:1019–30. doi: 10.1161/CIRCULATIONAHA.114.008788 PMC4367123 25779542

[166] Sano M Minamino T Toko H Miyauchi H Orimo M Qin Y . p53-induced inhibition of hif-1 causes cardiac dysfunction during pressure overload. Nature (2007) 446:444–8. doi: 10.1038/nature05602 17334357

[167] Yoshida Y Shimizu I Katsuumi G Jiao S Suda M Hayashi Y . p53-induced inflammation exacerbates cardiac dysfunction during pressure overload. J Mol Cell Cardiol (2015) 85:183–98. doi: 10.1016/j.yjmcc.2015.06.001 26055447

[168] Chen X Lin H Xiong W Pan J Huang S Xu S . p53-dependent mitochondrial compensation in heart failure with preserved ejection fraction. J Am Heart Assoc (2022) 11:e024582. doi: 10.1161/JAHA.121.024582 35656994 PMC9238719

[169] Sanders-van Wijk S Tromp J Beussink-Nelson L Hage C Svedlund S Saraste A . Proteomic evaluation of the comorbidity-inflammation paradigm in heart failure with preserved ejection fraction: Results from the PROMIS-HFpEF study. Circulation (2020) 142:2029–44. doi: 10.1161/CIRCULATIONAHA.120.045810 PMC7686082 33034202

[170] Gandhi PU Gaggin HK Redfield MM Chen HH Stevens SR Anstrom KJ . Insulin-like growth factor-binding protein-7 as a biomarker of diastolic dysfunction and functional capacity in heart failure with preserved ejection fraction: Results from the RELAX trial. JACC Heart Fail (2016) 4:860–9. doi: 10.1016/j.jchf.2016.08.002 PMC5500914 27744089

[171] Chaib S Tchkonia T Kirkland JL . Cellular senescence and senolytics: the path to the clinic. Nat Med (2022) 28:1556–68. doi: 10.1038/s41591-022-01923-y PMC9599677 35953721

[172] El Assar M Angulo J Rodriguez-Manas L . Oxidative stress and vascular inflammation in aging. Free Radic Biol Med (2013) 65:380–401. doi: 10.1016/j.freeradbiomed.2013.07.003 23851032

